# A prospective and consecutive study assessing short-term clinical and radiographic outcomes of Chinese domestically manufactured 3D printing trabecular titanium acetabular cup for primary total hip arthroplasty: evaluation of 236 cases

**DOI:** 10.3389/fsurg.2024.1279194

**Published:** 2024-03-27

**Authors:** Guo Chen, Chen Yi Wang, Zou Ma, Hong Lin Yi, Na Meng Bi, Wei Jiang Zhu, Jie Han, Sha Li Lu, Shang Shang Zhang, Hai Shen, Wu Hui Zhang, Peng Zhang, Yan Si

**Affiliations:** Department of Geriatric Orthopedics, Sichuan Provincial Orthopedic Hospital, Chengdu, Sichuan, China

**Keywords:** hip replacement, 3D printing technology, electron beam melting technology, porous titanium alloy, trabecular titanium acetabular cup

## Abstract

**Purpose:**

We prospectively evaluate the short-term clinical and radiographic outcomes of the only Chinese domestically produced trabecular titanium acetabular cup(3D ACT™ cup) in primary total hip arthroplasty (THA), aiming to provide evidence-based support for its clinical application.

**Methods:**

A total of 236 patients, who underwent primary THA using 3D ACT™ cup in the Department of Joint Surgery at our hospital between January 2017 and June 2019, were included in this study. General patient data, imaging information, functional scores, and complications were collected to evaluate the early clinical efficacy.

**Results:**

All patients were followed up for 33–52 months, with an average of (42.2 ± 9.2) months. At the last follow-up, the preoperative HHS score increased significantly from 43.7 ± 6.8 to 85.6 ± 9.3 points (*P* < 0.01). Similarly, the preoperative WOMAC scores showed significant improvement from 59.2 ± 5.8 to 13.1 ± 3.5 points (*P* < 0.01). 92.3% of the patients expressed satisfaction or high satisfaction with the clinical outcome. Furthermore, 87.7% of the acetabular cups were positioned within the Lewinnek safe zone, achieving successful reconstruction of the acetabular rotation center. The cup survival rate at the last follow-up was 100%.

**Conclusions:**

The utilization of the only Chinese domestically manufactured 3D printing trabecular titanium acetabular cup in primary THA demonstrated favorable short-term clinical and radiographic outcomes. The acetabular cup exhibits excellent initial stability, high survival rate, and favorable osseointegration, leading to a significant enhancement in pain relief and functional improvement. In the future, larger sample sizes and multicenter prospective randomized controlled trials will be required to validate the long-term safety and effectiveness of this 3D ACT™ cup.

## 1 Introduction

With the global aging population on the rise, there has been a steady increase in the incidence and prevalence of degenerative hip joint diseases, leading to a proportional surge in the number of patients requiring primary total hip arthroplasty (THA) (1–3). The non-cemented acetabular cup achieves initial stability through press-fitting with the host bone, leading to favorable long-term osseointegration or bone ingrowth. It has demonstrated good clinical outcomes and mid- to long-term survival rates in primary THA (4, 5). While surface manufacturing techniques like plasma spraying, high-temperature sintering, and electrophoretic deposition have been used to produce favorable surfaces for bone ingrowth in non-cemented cups, there have been numerous reports documenting failures attributed to debonding of sintered titanium beads and coating (6–8).

Three-dimensional (3D) printing technology has emerged in the past 20 years and is widely used in the field of orthopedic implant manufacturing. This technology uses three-dimensional model data as a basis and applies powdered metal or plastic adhesives to manufacture products with multi-level structures or complex geometric shapes (9). Electron beam melting (EBM) technology, a vital subset of 3D printing, presents a viable approach for fabricating trabecular titanium acetabular cups with desirable features including interconnected pore with high porosity and pore size for osseointegration, low elastic modulus, and a high surface friction coefficient. Studies have shown good bone-inducing and bone-conductive properties *in vitro* and in animal experiments (6, 10).

**Figure 1:**
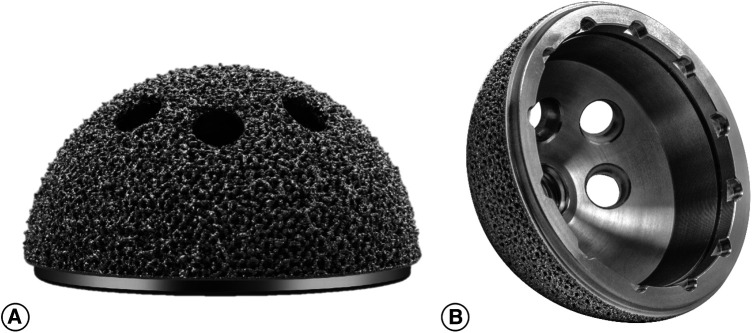
The picture shows the 3D ACT™ cup. The acetabular cup surface is distributed with interconnected-hole structure with high porosity. (**A**) Illustrates the lateral aspect of the acetabular prosthesis. (**B**) Illustrates the medial aspect of the acetabular prosthesis.

China had a delayed start in the research and development of 3D-printed acetabular cups. Currently, only one 3D printing trabecular titanium acetabular cup (3D ACT™ cup, Aikang, Beijing, China; Figure 1) is available on the market. Two retrospective studies have been conducted, involving 92 and 32 patients, reporting favorable short-to-medium-term clinical and imaging results of this Chinese domestically manufactured acetabular cup (11, 12). No prospective studies evaluating the clinical and imaging outcomes of this domestic cup in primary THA have been identified. In this study, we aimed to prospectively evaluate the clinical and imaging outcomes of this cup in primary THA through a relatively large sample size, in order to provide evidence-based support for the clinical application of the 3D ACT™ cup.

## 2 Materials and methods

### 2.1 General information

This study prospectively and consecutively collected patients’ basic information, laboratory and imaging results, and clinical functional scoring data with patients’ informed consent, in accordance with the Helsinki Declaration and approval from the hospital ethics committee. Patients who underwent primary THA in our hospital using 3D ACT™ cup from January 2017 to June 2019 in our hospital were prospectively and consecutively enrolled. The inclusion criteria required that the primary hip disease was hip osteoarthritis (HOA), femoral neck fracture (FFN), developmental dysplasia of the hip (DDH), avascular necrosis (AVN), rheumatoid arthritis (RA), ankylosing spondylitis (AS), or coxa valga (CV); and that patients were able to comply with follow-up for outpatient or telephone reviews. Exclusion criteria were: primary hip disease due to infection, tumor, or neurogenic arthropathy; severe lower limb vascular disease, severe dysfunction of major visceral organs, and patients with significant limb movement or sensory impairment due to severe spinal diseases. A total of 251 patients were enrolled in the study. One patient died due to cardiovascular accidents during follow-up, and 14 patients were lost to follow-up within one year after surgery. Ultimately, 236 patients (96 males [40.6%] with 96 hips and 140 females [59.4%] with 140 hips) were included in the study. The mean follow-up time was 42.2 months (range: 33–52 months). The mean age of the patients was 57.7 years (range: 32–89 years), and the mean body mass index (BMI) was 23.38 kg/m2 (range: 18.1–27.2 kg/m2). The patients’ primary diagnoses included 42 cases (17.8%) of HOA, 65 cases (27.5%) of AVN, 12 cases (5.1%) of RA, 78 cases (33.1%) of DDH, 26 cases (11.0%) of FFN, 7 cases (2.9%) of AS, and 6 cases (2.6%) of CV. The demographic data of the patients is shown in Table 1.

**Table 1:** Demographic characteristics of included cases.

Items	Cases (*n* = 236)	
Gender (*n*, cases)	Male	96
	Female	140
Age (x ± s, years)		57.7 ± 14.4
Body mass index (x ± s, kg/m^2^)		23.38 ± 3.9
Primary diseases (*n*, cases)	OA	42
	DDH	65
	RA	12
	AVN	78
	FFN	26
	AS	7
	CV	6
Surgery time (x ± s, mins)		56.1 ± 9.2
Intraoperative bleeding (x ± s, ml)		163.47 ± 46.78
Mean hospitalization time (x ± s, days)		7.5 ± 1.5
Follow-up time (x ± s, months)		42.2 ± 9.2

### 2.2 Prosthesis information, surgical procedure and postoperative management

In this study, 3D printing trabecular titanium(Ti6Al4V) acetabular cup (3D ACT™ cup, Aikang, Beijing, China) was used in all patients. The acetabular cup features a 1.5 mm thick porous coating with a porosity of 50%–80% and a pore size of 600–800 μm, enabling an interconnected pore structure. It exhibits an average elastic modulus of 0.5–1.3 GPa, similar to that of human cancellous bone. The friction coefficient between the cup and cancellous bone measures 1.08, whereas that between the cup and cortical bone is 0.93. The cup is available in 16 different sizes, ranging in diameter from 40 to 70 mm with a 2 mm increment between each size. The option of femoral stems primarily depends on the morphology of femoral medullary canal. A total of 158 cases utilized rectangular CL stems with titanium and hydroxyapatite coating, 25 cases employed cone-shaped MP stems with titanium coating for proximal fixation, 48 cases utilized wedge-shaped ML stems with titanium coating for proximal fixation, and 5 cases utilized modular SR stems with adjustable anteversion angle. All the aforementioned femoral stems were manufactured by Beijing Aikang Company. Bearing couplings were ceramic-on-ceramic in 53 patients (22.4%), ceramic-on- polyethylene in 146 patients (61.9%) and metal-on-polyethylene in 37 patients (15.7%).

All surgeries were performed by two experienced orthopedic surgeons(with more than 200 THAs per year individually). Both surgeons have undergone specialized training in the application of the 3D ACT™ cup and have collectively accomplished more than 150 THA utilizing this specific cup. The surgery was performed under general anesthesia following the standard protocol for THA through posterior-lateral approach described in literature (12). Patients were positioned in a lateral decubitus position, and the incision was made 1–2 cm posterior to the greater trochanter and 7–8 cm above it, with a length of 10–15 cm, bending from the starting point to the greater trochanter, and extending to the femoral shaft. The fascia lata was cut open to expose the tensor fasciae latae, and the gluteus maximus muscle fibers were separated. The piriformis and obturator internus muscles were cut, and the joint capsule was opened. The hip joint was dislocated by flexion and internal rotation. The femoral neck was cut off first, and the femoral head was removed. The next routine steps of THA were then followed. All acetabular cups were implanted using a 1 mm press-fit technique, and hip screws were implanted or not depending on the specific situation and the surgeon's experience. The joint capsule can be sutured or removed if necessary. The piriformis and obturator internus muscles were sutured, and the short external rotator muscles were reconstructed. Patients who did not undergo extrarticular osteotomy were not fitted with drainage tubes.

Postoperatively, patients were given diclofenac sodium tablets and intramuscular tramadol or dextromethorphan for pain relief. Other measures, such as antifibrinolysis, anticoagulation, nutritional support, prevention of nausea and vomiting, pain management, and blood management, were carried out according to the recommended guidelines for fast-track rehabilitation of hip and knee arthroplasty (13–16). On the second day after surgery, anteroposterior view of the pelvis and oblique view of the hip (or frog-leg x-ray) on the affected side were obtained and patients were guided to walk with the assistance of mobility aid.

### 2.3 Observational indicators

Patients are required to have follow-up visits at the outpatient department at 1, 3, 6, and 12 months after surgery, followed by annual visits thereafter. For patients who cannot come to the outpatient department for follow-up, their clinical scores are recorded through telephone follow-up, and x-rays are taken at other hospitals and mailed to the researchers.

#### 2.3.1 Clinical data

This includes patients’ general demographic data. We use the Harris Hip Score (HHS) (17), the Western Ontario and McMaster Universities Osteoarthritis Index (WOMAC) (18) to evaluate the clinical effects, and a self-assessment questionnaire for satisfaction (19) (divided into five levels: very dissatisfied, dissatisfied, neutral, satisfied, or very satisfied) to assess patients’ subjective satisfaction. The perioperative and postoperative complications (nerve damage, deep venous thrombosis, dislocation, periprosthetic infection, periprosthetic fracture, revision, etc.) is recorded. Survivorship estimation, with acetabular revision attributed to loosening as the endpoint, was performed using a Kaplan-Meier analysis.

#### 2.3.2 Imaging evaluation

**Figure 2:**
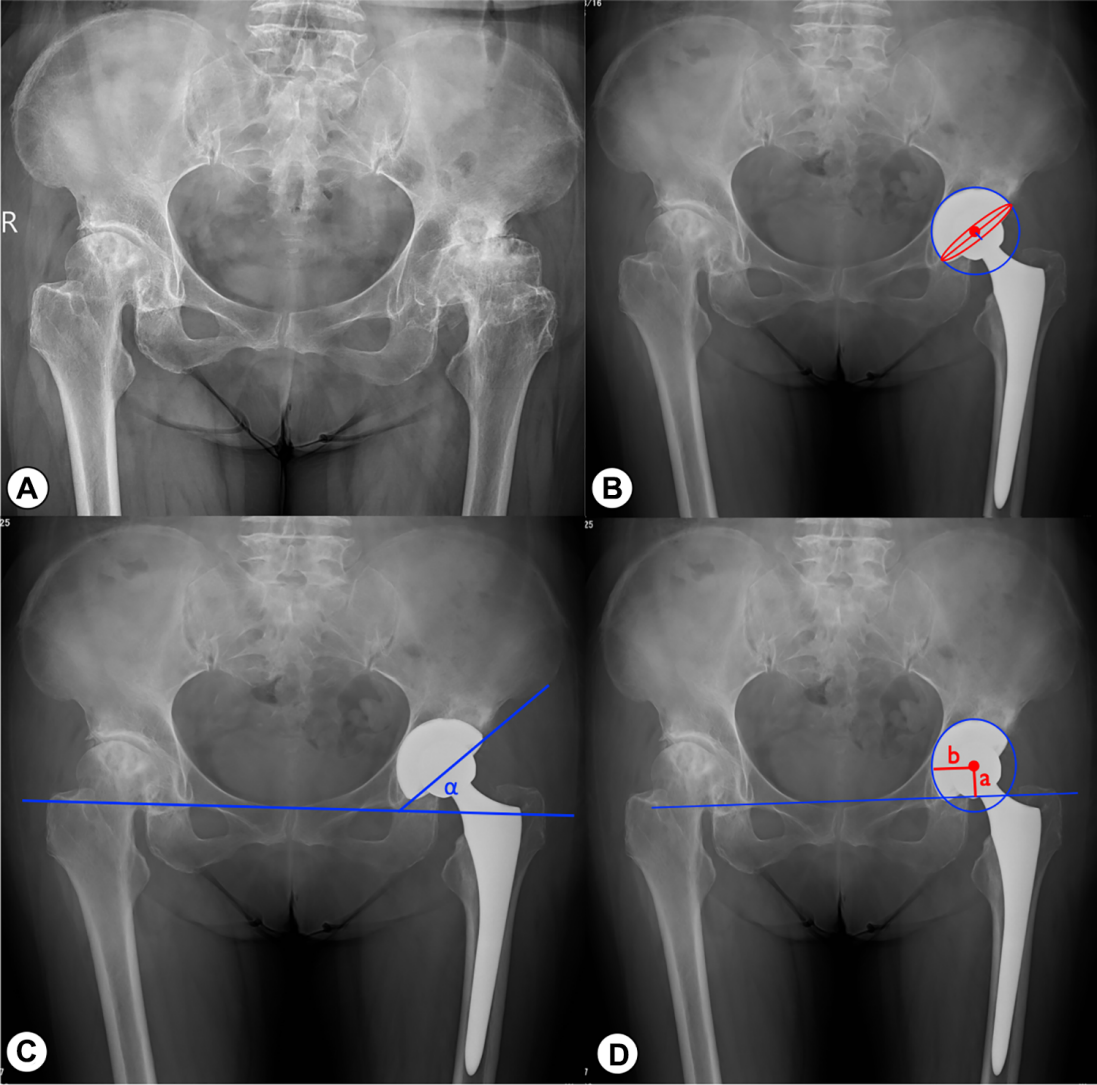
Imaging measurements on postoperative anteroposterior X-rays. (**A**) Preoperative radiograph of a patient with bilateral DDH. (**B**) Measure-ment of acetabular anteversion. Anteversion angle = arcsin(a/b); a is the minor axis of the red ellipse (the blue straight line in the figure); b is the major axis of the red ellipse (the red straight line in the figure). (**C**) Measurement of acetabular abduction angle. (**D**) The vertical rotation center (a) and the horizontal rotation center (b) of the hip joint.

Imaging evaluation involved the participation of three unbiased evaluators, including an orthopaedic surgeon, an orthopaedic resident, and a radiologist. These individuals independently conducted the measurements without any access to the patients’ clinical information. The imaging evaluation indicators include the following:

(1) Acetabular anteversion angle and abduction angle: On the anteroposterior pelvic x-ray, we measure the acetabular anteversion and abduction angles according to the method described by Lewinnek (20) (as shown in Figures 2A–C).
(2) The vertical and horizontal positions of the center of rotation (COR) (as shown in Figure 2D): The vertical distance of the COR (COR_ver_) is defined as the perpendicular distance from the center of rotation to the teardrop line (a). The horizontal distance of the COR (COR_hor_) is defined as the perpendicular distance from the center of rotation to the bottom of the teardrop (b). We compare the distance between the COR on the affected side and the healthy side in cases where the contralateral hip is a normal anatomical structure in order to reflect the COR recovery the operated hip.
(3) Cup loosening and bone integration: Evaluate the stability and bone integration of the acetabular prosthesis according to the criteria proposed by Moore et al. at the Anderson Orthopedic Research Center (21). Acetabular loosening is defined as: the gradual appearance of continuous radiolucent lines around the prosthesis, with a width greater than 2 mm; or acetabular prosthesis displacement greater than 5 mm or acetabular angle change greater than 5° after surgery.The criteria used to assess cup osseointegration consists of the following: (1) absence of radiolucent lines, (2) presence of superolateral buttresses, (3) presence of medial stress-shielding, (4) presence of radial trabeculae, and (5) presence of inferomedial buttresses. The assessment of stress shielding in (3) was based on the criteria outlined in Engh's study (22). Radiographic osseointegration is determined by the presence of at least three of these signs. The observer conducted radiographic analysis on each x-ray at least twice to ensure the accuracy of the assessments.

### 2.4 Statistical methods

All statistical analyses were performed using SPSS 26.0 software for Mac (SPSS Inc., Chicago, IL, USA) on personal computers. Continuous variables were expressed as means ± standard deviation, and t-tests were used for comparisons if they followed a normal distribution. If the data did not follow a normal distribution and/or exhibited heterogeneity of variance, Wilcoxon rank-sum tests were used instead. Chi-squared tests or Fisher's exact tests were used for categorical variables. A *P*-value less than 0.05 was considered statistically significant.

## 3 Results

### 3.1 Clinical data

The average operation time was (56.1 ± 9.2) minutes, and the average intraoperative blood loss was (163.47 ± 46.78) ml. Twelve patients (5.1%) received blood transfusions during or after surgery. The average length of hospital stay for all patients was (7.5 ± 1.5) days. During the perioperative period, 19 patients (8.1%) were found to have deep vein thrombosis in calf muscles before discharge, and after consultation with the vascular surgery department, anticoagulant therapy was adjusted, and follow-up was conducted in the outpatient department after discharge. Thrombus was found disappeared in four patients at 2 months after surgery, and the thrombus in the remaining 15 patients was also found to have disappeared during the follow-up at 3 months after surgery. Eleven patients (4.7%) had minor trochanteric fractures during surgery and were fixed with wires. Four (1.7%) DDH patients (Crowe II: 2 cases, Crowe III: 2 cases) with average abduction angle 39.5 ± 4.2° and anteversion angle 11.8 ± 2.4°) had hip dislocation during activity in the hospital after surgery and were treated by manual reduction. After repeated education to patients and their families on measures to prevent dislocation, no further dislocations occurred. No patients developed complications such as incision infection, delayed incision healing, periprosthetic infection, or prosthesis loosening until the last follow-up.

The HHS and WOMAC scores at 1 month and 1 year after surgery were significantly improved from the HHS scores before surgery(*P* < 0.01), while the HHS and WOMAC scores had no significant difference between 1 year after surgery and the last follow-up (*P* > 0.05). (see Table 2). At the last follow-up, 157 patients (66.5%) reported that they were very satisfied with their treatment, 61 patients (25.8%) were satisfied, 14 patients (5.9%) were neutral, and 4 patients (1.7%) were dissatisfied. None of the patients (0%) reported being very dissatisfied. Among the dissatisfied patients, one had DDH IV and underwent shortening osteotomy during surgery, resulting in a limb length discrepancy of about 2 cm on the operated side, requiring the use of shoe inserts. Another patient had AS with hip stiffness, and also had concomitant involvement of the spine and sacroiliac joints, with pelvic tilt and persistent limping at the last follow-up. The other two patients had RA with concomitant involvement of the knee and ankle joints, and their walking was affected by pain in other joints.

**Table 2:** Comparison of HHS and WOMAC scores at different follow-up time.

	Pre	Post-1m	Post-1y	Last follow-up	P1	P2	P3
HHS score	43.7 ± 6.8	70.6 ± 6.6	86.4 ± 11.3	85.6 ± 9.3	<0.01	<0.01	>0.05
WOMAC score	59.2 ± 5.8	25.2 ± 6.5	12.5 ± 3.4	13.1 ± 3.5	<0.01	<0.01	>0.05

P1: the comparison between 1 month after operation and preoperation; P2: the comparison between 1 year and 1 month after operation; P3: the comparison between the last follow-up and 1 year after operation. Pre, post-1m, post-1y respectively refer to preoperative, postoperative 1 month and postoperative 1 year.

### 3.2 Radiological results

**Figure 3:**
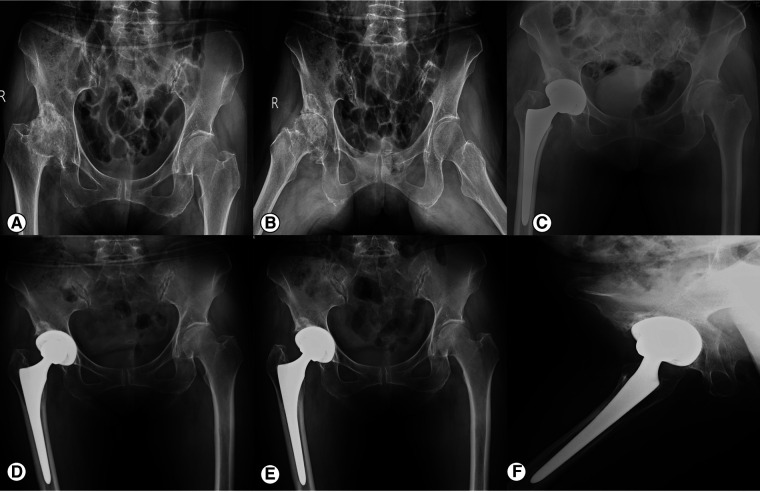
X-ray of a 61-year-old female DDH patient before and after THA. (**A, B**) The preoperative anteroposterior view of the pelvis and frog-leg X-ray; (**C**) The anteroposterior view of the pelvis on the first day after operation. The hip rotation center on the operated side has been restored, and the acetabular cup has a well anteversion angle and abduction angle. The femoral stem is implanted in the neutral position. (**D**) Anteroposterior X-ray of the pelvis 12 months after operation show that the prosthesis is well fixed. (**E, F**) The anteroposterior view of the pelvis and oblique view of the right hip at 48 months postoperatively. The prosthesis is well fixed and there is no lucent line between the acetabular prosthesis and the host bone.

**Figure 4:**
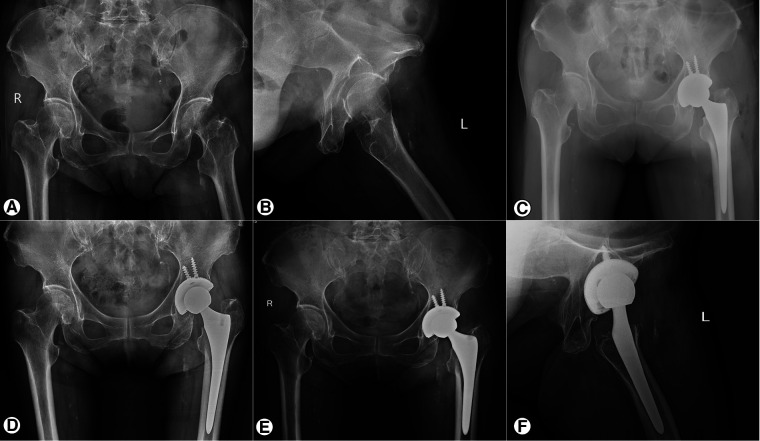
X-ray of a 73-year-old female FFN patient before and after THA. (**A, B**) The anteroposterior view of the pelvis and oblique view of the left hip before operation; (**C**) The anteroposterior view of the pelvis on the first day after operation. (**D**) Anteroposterior view of the pelvis 12 months after operation show that the prosthesis is well fixed. (**E, F**) The anteroposterior view of the pelvis and oblique view of the left hip at 42 months postoperatively. The prosthesis is well fixed and there is no lucent line between the acetabular prosthesis and the host bone.

The average acetabular cup abduction angle was (36.2 ± 4.2)°, and the anteversion angle was (18.8 ± 4.5)° on the operated side. According to the Lewinnek safe zone description(acetabular cup anteversion angle 15 ± 10 degrees, the abduction angle 40 ± 10 degrees), 87.7% (207/236) of the acetabular cups were located within this zone. The mean distance between the operated and healthy sides of COR_ver_ was (2.8 ± 0.5) mm, and that of COR_hor_ was (3.8 ± 0.7) mm. At the last follow-up, all cups showed good bone integration (at least three or more bone integration criteria met), and no cases met the criteria of cup loosening. In 18 cases (7.6%), a translucent line of 2 mm or less was observed between the acetabular cup and the bone bed during the one-month postoperative review, which disappeared within six months postoperatively. Four cases (1.7%) showed bone resorption in the femoral shaft mainly in Gruen zones (23) 1 and 14, but the patients had no clinical symptoms and were followed up for observation. Typical cases are shown in Figures 3, 4.

## 4 Discussion

Research on the design of acetabular coatings has become a hot topic in recent years. The coating manufactured by traditional techniques, such as ion spraying, high-temperature sintering, electrophoretic deposition, etc., can provide a certain degree of initial stability and long-term osseointegration between the cup and the acetabular bone. However, when dealing with cases with poor bone quality or quantity in primary THA such as osteoporosis, radiation-induced bone necrosis, and bone defects, the above-mentioned techniques may only provide limited initial stability. At the same time, some studies have reported galvanic effects of traditional cup and failures due to debonding of sintered titanium beads and titanium coating (6–8, 24). Traditional fabrication techniques usually have constraints on the design scheme, while 3D printing technology can manufacture acetabular cups with high porosity, appropriate and controllable pore size, elastic modulus similar to human bone, and better friction coefficient according to a three-dimensional model. The continuity between the porous and solid parts has been specifically developed to enhance resistance against corrosion and debonding (25). Perticarini (25) reported the clinical results of 3D printing trabecular titanium acetabular cup in European patients. The study included 133 patients, mainly diagnosed with HOA, AVN, and DDH. In at least 5 years of follow-up, all patients had significant pain relief and functional recovery. Castagnini (26) et al. conducted a retrospective study of consecutive cases, including 24 cases with an average follow-up of 79 months. The main hip diseases involved were not specified in the article. Their report confirmed the reliable mid-term clinical and radiographic results of this cup. Our study prospectively included more cases, with DDH (33.1%), AVN (27.5%), and HOA (17.8%) accounting for 78.4% of the total. We discovered that during the average follow-up period of 42.2 months, all 236 acetabular cups achieved good initial stability and long-term bone osseointegration with significant improvement in hip function on the operated side.

The good clinical and radiographic results of 3D ACT™ cup depend on its good initial stability and long-term bone ingrowth. In primary THA, the initial stability of the traditional coated cup will be significantly affected when encountering patients with advanced age, osteoporosis, metabolic bone disease, acetabular retroversion, RA, DDH, and other conditions associated with poor bone quality and quantity, and inadequate acetabular coverage (27–29). According to Mühlenfeld's research (30), rheumatoid arthritis (RA) is a bone-loss disease. The majority of patients with RA also have osteoporosis, and the medications used for the RA treatment can affect the osseointegration of non-cemented implants. The incidence of DDH is high in underdeveloped regions of China. Literature reports indicate that more than 50% of these patients may not be suitable for the use of traditional coated non-cemented acetabular cups due to the loss and weakening of the subchondral bone on the acetabular side (31). In hip revision surgeries, it is more common to encounter patients with poor bone quality and quantity (32, 33). In such circumstances, the utilization of the 3D printing trabecular titanium cup presents unique advantages and has been reported in the literature to yield positive outcomes (34). Our research was conducted in an economically underdeveloped region in Southwest China, where the incidence rate of DDH is high. In this study, DDH patients accounted for the highest proportion. The Chinese domestically produced 3D ACT™ cup has also yielded favorable outcomes in this patient population.

Favorable clinical and radiological outcomes of the 3D printing trabecular titanium acetabular cup are dependent on its excellent initial stability and long-term osseointegration, which may be related to the following factors. Firstly, the cup's high porosity, pore size, and a structure with interconnected pores in a three-dimensional arrangement provide good friction between the cup and the host bone (26). Castagnini's research (26) reported that the friction coefficient between the cup and cancellous bone is 1.08, which is better than porous tantalum (0.98) and sintered titanium beads (0.5). A good friction coefficient provides good initial stability for the cup and also is beneficial to long-term bone ingrowth. Secondly, the porous structure reduces the elastic modulus of the cup and can change the strength and mechanical properties of it by adjusting the pore size and porosity (35, 36). The elastic modulus of the cup is on average 0.5–1.3 GPa, which is similar to human cancellous bones. It facilitates efficient load transfer, mitigates the risks associated with stress shielding and bone resorption, and enhances the rate of bone integration (37). Furthermore, The cup's highly porous and pore-interconnected structure closely mimics the natural trabecular bone architecture. Researches by Ponader (38) and Otsuki (39) have confirmed that high porosity is conducive to improving local blood flow and vascular formation, reducing fibrosis of the surrounding tissue of the implant, and stimulating new bone growth into the pores. Similarly, research by Devine (40) has also confirmed that the aforementioned characteristics of the cup have bone-inducing and bone-conducting properties. The above characteristics of the cup provide a good foundation and necessary conditions for long-term bone ingrowth. This has also been confirmed in the study by Perticarini's and ours. Perticarini (25) reported that no radiolucent lines or cup migration were observed during the follow-up. In our study, all cups showed excellent bone integration without any radiolucent lines at the last follow-up. Currently, in the initial THA procedures conducted abroad, acetabular cups utilized include the Depuy company's Pinnacle Gription acetabular cup, Biomet company's Regenerex Porous Titanium Construct acetabular cup, and Stryker&Zimmer company's Trabecular Metal Modular Acetabular System acetabular cup, all prepared through processes such as sintering and low-temperature arc deposition. In comparison with the aforementioned mainstream foreign acetabular cup products, the 3D printing trabecular titanium acetabular cup exhibits characteristics of more controllable porosity and pore structure during fabrication. Its increased surface roughness provides greater frictional strength, enhancing the initial stability of the prosthesis. The biomimetic trabecular structure facilitates superior mechanical conduction and long-term bone ingrowth.

The position of cup is one of the critical factors for postoperative hip joint stability in THA. In this study, 87.7% of the cups were placed within the Lewinnek safe zone with low dislocation rate (41). Despite some studies questioning the role of the Lewinnek safe zone (42), many literature still considers the cup placement within this zone as the most important predictive factor for postoperative dislocation. Another important factor in preventing dislocation is the restoration of the hip's offset. The study conducted by Vaishy et al. highlights the significance of concurrent reconstruction of both horizontal and vertical offset to minimize dislocation risk and improve the hip range of motion (43). In our study, successful reconstruction of the rotational center of the operated hip was achieved, thereby restoring the offset and soft tissue tension to a satisfactory extent and reducing the dislocation rate. These 4 patients, who experienced dislocation, are all diagnosed with DDH(Crowe II: 2 cases, Crowe III: 2 cases), and the anteversion angle of the acetabular cups was relatively small (average 11.8 ± 2.4°). It is reasoned that this could be attributed to the insufficient bone stock in the acetabulum of DDH patient, leading to a smaller anteversion angle placement to enhance the coverage of the acetabulum during surgery. This could be the cause of hip dislocation in these four patients.

Although this study reported good early clinical and imaging results, as well as patient satisfaction in 236 cases of 3D ACT™ cup, it still have some limitations. Firstly, this is the only Chinese-manufactured 3D printing trabecular titanium acetabular cup that can be used clinically and this study is the most extensive prospective research found to date, with the highest patient enrollment that can be retrieved of 3D ACT™ cup. Even so, the follow-up duration was relatively short, and a control group was not established due to factors such as prosthetic supply of our hospital during that period. Further large-scale, long-term prospective randomized controlled trials are needed to confirm its safety and efficacy. Secondly, there were some lost to follow-up cases, which may have some impact on the results. Thirdly, the surgeries in this study were performed by two senior orthopedic surgeons, but they may also have some confounding effects on the results. Finally, in the current study, evaluations such as CT three-dimensional reconstruction and bone density DXA were not performed, and the serum metal ion concentration was not detected to evaluate the corrosion safety of 3D ACT™ cup. In light of these limitations, it's important to acknowledge that the study's findings may primarily pertain to the short-term scenario and may not fully represent the broader spectrum of challenges and outcomes that could emerge over an extended period. Consequently, when applying the conclusions, one should be cautious and recognize the study's constraints in addressing the comprehensive and nuanced aspects of the clinical context.

## 5 Conclusions

The use of the only Chinese domestically manufactured 3D printing trabecular titanium acetabular cup in primary THA demonstrated favorable short-term clinical and radiographic outcomes. The acetabular cup exhibits excellent initial stability, high survival rate, and favorable osseointegration, leading to a significant enhancement in pain relief and functional improvement. In the future, larger sample sizes and multicenter prospective randomized controlled trials will be required to validate the long-term safety and effectiveness of the 3D ACT™ cup.

## Data Availability

The raw data supporting the conclusions of this article will be made available by the authors, without undue reservation.
